# *‘*It is empowering and gives people dignity in a very difficult process’: A multistage, multimethod qualitative study to understand the views of end users in the cultural adaptation of a dementia and driving decision aid

**DOI:** 10.1111/hex.14006

**Published:** 2024-03-18

**Authors:** Nadine Veerhuis, Alessandra Merizzi, Stephanie Papoulias, Claire Bradbury, Kathy Sheret, Victoria Traynor

**Affiliations:** ^1^ Aged, Dementia, Health Education and Research Centre, Faculty of Science Medicine and Health, School of Nursing University of Wollongong Wollongong New South Wales Australia; ^2^ Memory Assessment and Treatment Service Pennine Care National Health Service Foundation Trust Oldham UK; ^3^ Memory Assessment Service Dorset Healthcare University Foundation Trust, Alderney Hospital Poole UK; ^4^ Present address: Research Department, Centre for Socio‐Economic Research on Aging National Institute of Health and Science on Aging (INRCA) Via Santa Margherita, 5 Ancona 60124 Italy; ^5^ Present address: Sheffield Health and Social Care NHS Foundation Trust, Fulwood House Old Fulwood Road Sheffield S10 3TH UK

**Keywords:** Alzheimer's disease, decision aid, dementia, driving, person‐centred, shared decision making

## Abstract

**Background:**

Decisions about driving for individuals living with dementia (ILWD) can be challenging. There are limited evidence‐based person‐centred interventions in the United Kingdom that support decisions about transitioning to not driving or guidelines for developing decision aids for ILWD. This study aimed to understand the important features of a decision aid through the cultural adaptation of Australian dementia and driving decision aid (DDDA) for ILWD residing in the United Kingdom.

**Methods:**

This qualitative study was theoretically underpinned by a person‐centred framework and conducted over three stages: (1) Development of a draft UK‐specific DDDA; (2) semistructured interviews with ILWD and an online survey with stakeholders to obtain their views on a draft UK DDDA and (3) content analysis and synthesis of qualitative data to inform the final version of the decision aid.

**Results:**

Eleven ILWD and six of their spouses participated in interviews, and 102 stakeholders responded to an online survey. The four broad features identified as important to include in a decision aid for drivers living with dementia were: a structured and interactive format; positive and supportive messaging and presentation; relevant and concise content and choice‐centred. The perceived benefits of the decision aid were identified as supporting conversations, enhancing collaborative decision making and enabling agency with decisions about driving and future mobility options.

**Conclusions:**

Decision aids that are underpinned by interactive choice‐driven questions enhance a person‐centred approach to decisions about driving. Positively framing decision aids through the presentation and content can facilitate engagement with the decision‐making process about driving. The findings have implications for the development of decision aids designed for ILWD on other important health and social topics.

**Patient and Public Involvement:**

Advocating for the development of a UK DDDA were ILWD. Healthcare professionals contributed to the development of a draft UK DDDA. Former and current drivers living with dementia, family members, healthcare professionals and other support networks of ILWD participated in interviews or an online survey which informed the final version of the UK DDDA.

## 1 INTRODUCTION

In the United Kingdom, one in three people who are living with dementia continues to drive a car.^1^ Although living with dementia does not necessarily immediately exclude an individual from driving,^2^ dementia, such as Alzheimer's disease, is a progressive condition making the transition into not driving inevitable.^3^ Decisions about when to adopt alternatives to driving are, however, not straightforward because of the unique variation in how individuals living with dementia (ILWD) experience health declines and are negatively impacted after cessation of driving.^4^, ^5^, ^6^ Discussing and planning for this transition early in the decision pathway is a clinically supported approach advised in driving guidelines across various countries.^7^, ^8^, ^9^ Providing accessible, person‐centred information promptly can assist with decisions and prepare for this life transition, yet few resources exist.^10^, ^11^

Similar to other developed countries, in the United Kingdom, the final decision to renew an individual's driving licence is made by the driving licensing authority.^2^, ^7^, ^12^ These decisions are made in consultation with medical practitioners, who are obligated to communicate the legal requirements about driving with an individual living with dementia upon their diagnosis.^2^ Nevertheless, medical practitioners identify numerous challenges when assessing and discussing driving with these individuals.^13^, ^14^

Despite this, there are several reasons why the opinions of ILWD are crucial in any decision about driving. First, the views of their key informants might differ from those of the ILWD.^6^ Second, driving is perceived by the ILWD as key to sustaining independence and identity.^6^ Third, ILWD desires involvement in decisions about driving.^5^ Additionally, ensuring that ILWDs are central to decisions about their health and social decisions is fundamental to person‐centred care, which is integral to high‐quality clinical health practice.^15^ Despite the importance of inclusion and shared decision‐making about driving with ILWD, family members and healthcare professionals identify driving as a challenging and delicate topic to raise.^16^

Decision aids such as booklets or online tools support a person's ability to participate in decisions by providing information about the risks and benefits of various options.^17^ Increased knowledge, enhanced communication and participation in decisions, and reduced decisional conflict are demonstrated benefits of using decision aids for ILWD for a range of health decisions.^18^ For decision aids developed for ILWD in written format, variation occurs in how they are presented (for example, see Ottawa Hospital Research Institute, 2019). Therefore, understanding the views of ILWD and their support networks on the preferred format of decision aids is needed.

Engaging end‐user views in development stages ensures decision aids meet the international quality standards.^19^ However, limited studies detail the process of engaging end‐user views.^20^ In addition, the family member or substitute decision maker is often engaged in the development process, rather than the ILWD^18^ or the target group.^21^ Two studies tested the effects of Australian dementia and driving decision aid (DDDA) with ILWD and explicitly sought these individuals' feedback.^22^, ^23^ After using the decision aid, drivers living with dementia showed improvements in knowledge about dementia and reduced decisional conflict about driving.^22^, ^23^

Although the evidence supporting the outcomes of using decision aids more broadly is strong,^24^, ^25^ there are challenges with developing decision aids from the outset. Costs and time constraints make culturally adapting existing decision aids a potentially cost‐effective alternative.^26^ Ensuring decision aids are culturally and linguistically appropriate in a different cultural context is crucial,^27^ as there are differences between countries with similar cultures regarding the perceived usefulness of decision aids and how people make decisions.^28^

The cultural adaptation of digital health tools is considered important to improving accessibility, however, it has been inadequately reported and an understanding of the nuanced approaches for groups with ‘specific distinctions’ is required.^29^, p.1 Similarities can be found in the components of adaption across various contexts including health interventions more generally,^30^ health behavioural interventions,^31^ digital mental health tools,^32^ health communication resources^33^ and decision aids.^27^ Piloting and reviewing the intervention or tool by including the views and opinions of end‐users is highly recommended so that the decision aid is acceptable and tailored to their needs.^27^, ^31^, ^33^ To date, empirical research describing the cultural adaptation of decision aids for ILWD is limited.^34^, ^35^

This study originated with two authors who facilitated support groups for ILWD in England, United Kingdom, where the challenges of transitioning into not driving emerged. ILWD highlighted the lack of clarity about driving licence responsibilities, the licensing renewal process, and the lack of information and support when cessation of driving was imminent. The support group members voiced a desire to develop a UK DDDA after viewing an Australian DDDA. The Australian DDDA is presented in four colour‐coded steps providing information about: (1) dementia, driving, options and warning signs; (2) decisional needs, values and support; (3) benefits and risks of driving and (4) further guidance and making the decision. The theoretical framework and process to develop the DDDA is described elsewhere.^22^ The DDDA is freely available online at www.adhere.org.au. Providing this brief historical excursus highlights the bottom‐up approach from which the UK DDDA study originated.

This study was a collaboration between the University of Wollongong, Australia, and Pennine Care National Health Service Foundation Trust, England. The study aimed to engage the views of ILWD and end users in the cultural adaptation of an Australian DDDA to create a UK DDDA. To achieve this aim, the following research questions were answered:

1. What features of the draft UK DDDA are important to ILWD and stakeholders involved with decisions about driving?
2. What amendments are required to the draft UK DDDA version to ensure a new version of the decision aid is relevant and applicable to UK drivers living with dementia?

## 2 METHODS

### 2.1 Study design

This qualitative, multimethod study^36^ using an online survey and interviews, was theoretically guided by the process elements emphasised in the person‐centred framework developed by McCormack and McCance,^37^ ^,p.42^: the importance of shared decision making, engaging authentically with the individual living with dementia, and understanding the person's core values. These elements informed the chosen method and recruitment strategy to engage ILWD, the analysis process and decisions to amend the final decision aid to inform a UK DDDA.

**Figure 1:**
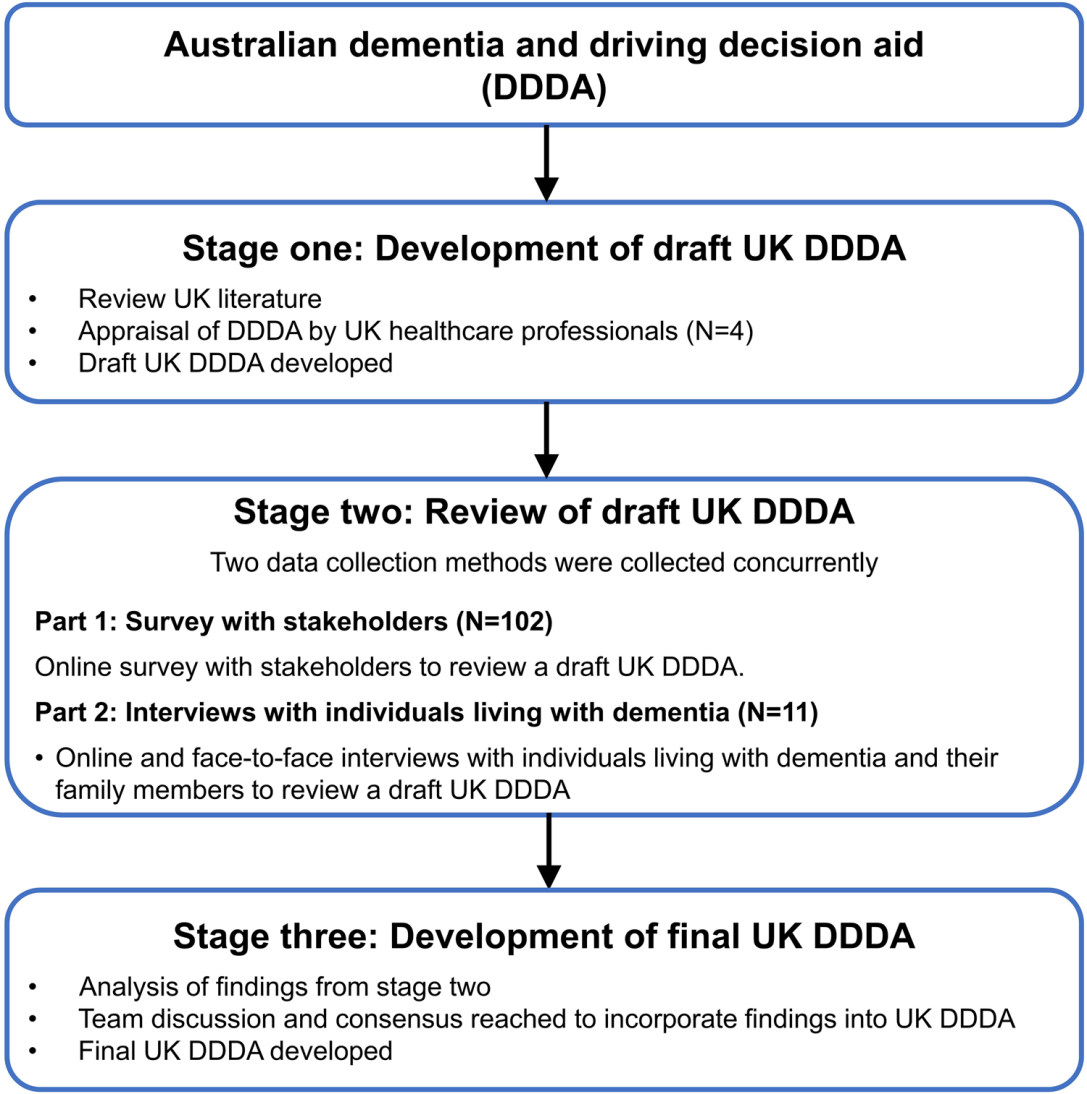
Cultural adaptation process.

Specific data collection and recruitment methods were employed to ensure the views of ILWD were gathered and represented in this study. The UK research team were professionals registered in psychology and nursing and had extensive specialist experience working with ILWD. The study was conducted between June 2019 and November 2021 in three stages (see Figure 1). A draft UK DDDA was produced in Stage 1. In Stage 2, an external review of the draft UK DDDA was conducted using a concurrent two‐part process: Part 1 included a qualitative online survey with stakeholders, and Part 2 employed interviews with current and retired drivers living with dementia. The findings from the analysis of the interview and online survey data informed amendments to the draft UK DDDA in Stage 3, to create a final version of the UK DDDA.

Ethical approval to undertake the study was provided by the UK National Health Research Authority (IRAS Project ID: 255012) and the University of Wollongong Human Research Ethics Committee (2019/3735) before data collection commenced. The reporting of this study follows the Consolidated Criteria for Reporting Qualitative Research for the interviews conducted^38^ (see Supporting Information S1: Appendix A). The DEVELOPTOOLS checklist was used for reporting user involvement in the cultural adaptation of the decision aid^20^ (see Supporting Information S1: Appendix B).

### 2.2 Stage 1: Development of a draft UK DDDA

To start, a literature review on UK‐specific dementia and driving regulations, statistics and guidelines were undertaken to identify amendments required to the Australian DDDA. Relevant information from the literature review and feedback from four UK healthcare professionals (one from the UK research team) were incorporated into the draft UK DDDA by a desktop publisher (a professional paid to assist with the presentation and layout of the decision aid). The draft UK decision aid was created in a portable document format (PDF) and participants in Stage 2 were provided with a printed hard copy of the resource or an online web version, accessible by a web link. Similar to the Australian DDDA, the draft UK DDDA was a 32‐page PDF consisting of four colour‐coded steps: (1) to help clarify my decision, (2) what do I need to make my decision, (3) weighing my options and (4) what next? Further details about the development and theoretical underpinnings of the original Australian DDDA have been described previously.^22^

### 2.3 Stage 2: External review of the draft UK DDDA

#### 2.3.1 Participants and recruitment

The engagement of ILWD and stakeholders was undertaken concurrently in two parts. In Part 1, a convenience sample, using snowballing recruitment^39^ of healthcare professionals, family members and ILWD, was recruited. In Part 2, purposive sampling^40^ was used to invite ILWD to provide their views and opinions about what was useful and what needed to change in the draft UK DDDA to ensure the final UK version would be relevant and acceptable.

##### Part 1: Qualitative online survey

Inclusion criteria for completion of the qualitative online survey were current or retired drivers who self‐identified as having dementia or memory loss, healthcare professionals who worked with ILWD, carers or a family member or friend of an individual living with dementia or memory loss. Participants were 18 years or over, able to read and write in English, lived in the UK and had access to the internet to complete the qualitative online survey. The survey sought views on what respondents liked and what they would change to the UK DDDA (to view the survey questions see Supporting Information S1: Appendix C). A range of strategies was used to recruit participants, including email invitations, social media, word of mouth and advertisements through several channels, including the Aged Dementia Health Education and Research group, government‐run memory clinics, UK healthcare professional networks, community associations and organisations that support ILWD. Participants self‐nominated by clicking on the link to the online survey, which contained an information sheet about the study. Tacit consent was obtained through the completion of the survey.

##### Part 2: Online and face‐to‐face interviews with current or retired (former) drivers living with dementia

The Pennine Care NHS Foundation Trust was the primary recruitment avenue to invite drivers or retired drivers living with dementia to take part in an interview. Invitations and promotional materials were sent by post or email to managers of Pennine Care Memory Assessment and Treatment Service who then informed eligible service users about the opportunity to participate in this research. Potential participants then contacted the researchers by email or telephone confirming their willingness to participate. Hard copies of the printed version of the draft UK DDDA, information sheet and consent form were mailed to each potential participant and an interview time was arranged. Written and/or verbal consent was obtained before each interview commencing.

Participants were 18 years and over, self‐identified as living with dementia or memory loss, could read and understand the participant information sheet and consent form and, if unable to provide written consent, could provide verbal consent. A psychologist and assistant psychologist (A. M., S. P.) with postgraduate qualifications and extensive experience working with ILWD facilitated the interviews. The communication software Zoom (Zoom Video Communications Inc.) is a convenient and cost‐effective alternative to face‐to‐face interviews and due to the COVID‐19 pandemic was used to conduct and record some of the interviews online.^41^ A gift card for the value of 30 pounds was provided to participants as reimbursement for their time.

#### 2.3.2 Data collection

##### Part 1: Qualitative online survey

The online consultation was undertaken over 10 months using the online survey software, SurveyMonkey®. The research team developed the online consultation questions, which included four demographic questions and four open‐ended questions. Using skip logic, there were an additional five demographic questions for ILWD and one for professionals. The open‐ended questions included: ‘What did you like about the booklet?’, ‘What would you change about the booklet?’, ‘Would you like to review each page of the booklet?’ and ‘Do you have any final suggestions or comments to make in general about the booklet’. There were 17 additional questions for participants who wished to review each page of the booklet.

##### Part 2: Online or face‐to‐face interviews with drivers and retired drivers living with dementia

Eleven semistructured interviews with older drivers living with dementia were conducted over 2 months between August and September 2020. Interviews ranged from 20 to 60 min in duration, were conducted at a mutually convenient time and were audio recorded. There were no participant withdrawals. The interviewers took notes during and after each interview. Six open‐ended, semistructured questions guided the interview schedule (Table 1).

**Table 1:** Semistructured interview questions.

1. What did you like or find useful about the booklet? 2. What changes should we make to the booklet to make it relevant and useful for those living in the UK? 3. Would you add anything to the booklet? 4. How would you use the booklet; on your own, with a family member or with a healthcare practitioner? 5. What format would you like to use the booklet? 6. Who would you like to receive the booklet from?

### 2.4 Data analysis

Demographic profiles were summarised using Microsoft Excel, version 16 (Microsoft Corporation). Three members of the research team transcribed audio files from the interviews into Word documents. Due to time restraints and the appropriateness of returning transcripts to participants with ILWD, this was not included in the ethical protocol. Any identifying information from data collected through transcripts and online surveys such as names or email addresses were anonymised before analysis. Transcripts from the interviews and responses from the online survey collected in SurveyMonkey® were imported as separate Word files and an Excel file into NVivo (QSR International), the software tool used to facilitate inductive qualitative content analysis of the data in a two‐phase process. Qualitative content analysis is a method to analyse data from various sources and describe new knowledge.^42^

First, using two priori codes to differentiate the positive feedback from suggested changes, three researchers conducted content analysis with the interview transcripts and one researcher with the online survey data. The research team then reviewed, reflected upon, and discussed each suggested change until a consensus was reached about the inclusion of the suggested amendment.

A secondary analysis, again using inductive qualitative content analysis, was conducted with both datasets to gain knowledge and insights to understand important features of a decision aid for drivers living with dementia.^42^ With the interview transcripts, one author (N. V.) became familiar with the whole data set by listening to the interviews, reading the transcripts and making notes. Meaningful units (words or a sentence) were then highlighted through open coding and labelled.^42^ Data saturation was considered to be reached when no new codes were identified. Categories were identified, then reviewed and refined based on similarities and differences between codes. Themes were then generated from the categories and exported into a Word table with corresponding quotes.^43^

The coding process was repeated for the free text responses from the online survey. Once the themes from Part 1 and Part 2 were identified, they were reviewed and refined by the research team until agreement was reached about labels and alignment with the categories.^42^ A synthesis of the themes identified in Part 1 and Part 2 was conducted and mapped onto a Word table. A comparison was undertaken to identify similarities and differences between the interviews with ILWD and online survey findings which informed any final amendments to the decision aid.

## 3 RESULTS

To ensure the views of ILWD are explicit, the presentation of the following demographic findings is distinguished from the stakeholder participants. Then a synthesis of the themes from the qualitative data is presented, highlighting any differences or commonalities between the views of ILWD and the other stakeholders.

### 3.1 Part 1: Qualitative online survey

A total of 160 participants entered the survey. Applying snowballing recruitment prevented the survey participation rate from being determined. The survey completion rate was 64%, determined by the completion of one or more open‐ended questions and demographic questions. One participant was not a resident of the United Kingdom, so that person's responses were removed from the analysis. A total of 102 surveys were retained for the analysis.

Participant demographics for the online survey are described in detail in Table 2. Most participants were female (81%), were aged 30 to 49 years and were professional carers or healthcare professionals (76%). Three ILWD took part in the survey, and nearly half (48%) of all participants reviewed each of the 32 pages of the UK DDDA. Half (50%) of the participants did not suggest any amendments to the draft UK version.

**Table 2:** Participant characteristics of the qualitative online survey.

Demographic category	Number (*N*)	Percentage (%)
Gender		
Female	83	81
Male	19	19
Age (years)		
18–29	9	9
30–49	45	44
50–64	42	41
65–74	4	4
75–84	1	1
85 and over	1	1
Location		
England	93	91
Wales	7	7
Scotland	1	1
Not disclosed	1	1
Identified role		
Professional carer/healthcare professional	78	76
A family member or friend of an individual living with dementia	10	10
or memory loss		
Other^a^	11	11
Individuals living with dementia or memory loss	3	3
The profession of healthcare professional^b^		
Nurse	28	36
Dementia support worker	11	14
Dementia advisor	10	13
Psychologist	9	12
Occupational therapist	8	10
Other	8	10
Psychiatrist	3	4
Social worker	1	1
Individuals living with dementia		
Driving years, mean (SD)	32 (±4)	
Living arrangements		
With spouse/partner in the family home	3	100
Education		
Lower secondary school	1	33
Upper secondary school	1	33
Master's degree	1	33
Driving frequency		
Once a week	1	33
2–6 times per week	1	33
More than once a day	1	33
Access to public transport		
Poor	1	33
Very poor	2	67
What did your like about the booklet?		
I liked…	93	91
No comment	9	9
Number of participants who reviewed each page of the booklet		
Yes	49	48
No	53	52

^a^ Other includes: researcher, dementia charity worker, policy officer, dementia service administrator and transport industry representatives.

^b^ Percentage calculated from healthcare professional sample.

### 3.2 Part 2: Online and face‐to‐face interviews with drivers or retired drivers living with dementia

Eleven ILWD took part in online interviews, six (55%) were interviewed with their spouses, and all but one spouse was female. All participants lived in England. Most were current drivers (*n* = 7, 64%), male (*n* = 8, 73%) and aged between 67 and 86, with a mean age of 76 years (SD ± 6).

### 3.3 Synthesis of qualitative findings of the free text responses from the online survey and interviews

The participants overwhelmingly welcomed the development of the UK version of the DDDA. ILWD perceived that the UK DDDA ‘made you think about your driving capabilities’ and indicated they would use the UK DDDA in a range of ways, together with family, a friend, their general practitioner, within a dementia support group or memory service or would review the resource only by themselves. Memory clinics and general practitioners were perceived to be the most accessible and effective pathways to obtain a copy of the final UK DDDA.

For stakeholders, the topic of dementia and driving was perceived as an important issue. The absence of a resource addressing this topic was highlighted, and the concept and availability of a decision aid for drivers living with dementia was viewed positively. Despite the resource being developed for ILWD, stakeholders indicated that a UK DDDA would provide benefits through enhanced collaborative decision‐making, initiating conversations and empowering ILWD to have agency in the decision‐making process. Various situations were identified by stakeholders in the way they would use the decision aid such as during fitness to drive assessments, before or after a diagnosis of dementia. Family members found the resource useful and ‘thought the booklet was really useful and helpful’ (Female, family member of an individual living with dementia, 65–74 years).

For some healthcare professionals, driving was a sensitive and complex topic to broach with the ILWD and therefore the resource would help with the decision process, make these conversations easier to initiate, and avoid the situation where a licence is forcibly removed. As one nurse described:

It is the area I find most difficult when talking to my patients and I would definitely want to use this leaflet to help them to make a decision. It is often a contentious subject and due to the rural nature of my case load (and the lack of public transport) patients are often very defensive about their right to continue driving… This leaflet would be an excellent tool for addressing this. (Female, nurse, 75–84 years)

The interdisciplinary utility of the decision aid to various healthcare professionals including nurses, general practitioners, dementia support workers, dementia advisers and psychologists, was identified. Healthcare professionals indicated that decision aid would be useful in a clinical practice setting as described by the following comments:

… it might be used to stimulate discussion, taking some ‘heat’ out of the decision‐making process. (Female, occupational therapist, 50–64 years)

[I] think it will be a very valuable resource when I am conducting my assessment visits, before and/or after diagnosis. (Female, registered nurse, 50–64 years)

Conversely, there were a small number of participants who identified barriers to using the decision aid in the future. These barriers included the decision aid being too long and the ILWD may lack insight into their driving ability, therefore the decision aid may not be useful to those individuals. Furthermore, although health professionals, a spouse and an ILWD perceived the decision aid would enhance collaborative decisions about driving, one ILWD was unsure how the decision aid would assist her to reach a decision about driving.

I don't understand how you use your answers to make a decision on driving. (Female, 50–64 years, driver living with dementia)

#### 3.3.1 Important features for a UK DDDA

**Figure 2:**
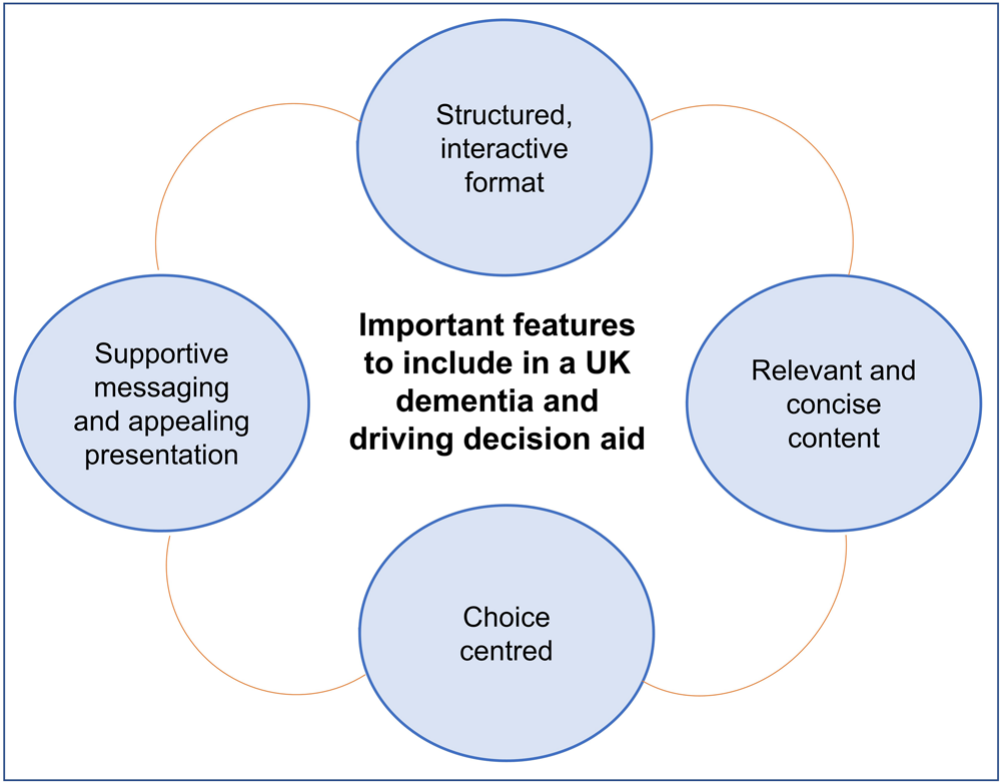
Themes representing important features of dementia and driving decision aid.

Four themes were identified that describe important features to maintain in the final UK DDDA version. These features include a structured and interactive format, supportive messaging and appealing presentation, relevant and concise information, and choice centred (see Figure 2). We outline in the following narrative any perceived differences between the interview transcripts for ILWD and the analysis of the stakeholder survey responses.

##### Structured and interactive format

Stakeholders identified that the draft UK DDDA engaged a person‐centred approach to decision making and this was perceived as a crucial feature. A structured, step‐by‐step and interactive format, involving checklists, options or open‐text responses, facilitated an individualised, person‐centred approach. The benefits for ILWD, with these features included in the decision aid, were described by one dementia care adviser:

… like how it enables a person with dementia to make a decision themselves about driving. It is empowering and gives people dignity in a very difficult process. (Female, dementia adviser, 30–49 years)

Most stakeholders and ILWD perceived these to be a valuable feature of the draft UK DDDA, as this format facilitated a sense of control and empowerment for the ILWD in the decision process. The UK decision aid was judged as balanced and ‘not pushing you’ to decide one way or another. An individual living with dementia described how this balanced approach was valued:

They have tried to not make the decision for you or influenced that. They have just given you the information and saying [sic] you know this is what it is. I didn't think you could improve on that. (Female, retired driver living with dementia, 82 years)

##### Supportive messaging and appealing presentation

The interactive person‐centred approach also contributed to overall perceptions that the decision aid would support the decision making process. The inclusion of positive images, provision of alternatives to driving, signposting to further support and balanced content contributed to the overall perceptions of a positive supportive aid. For example, one spouse of an ILWD commented:

It's not all doom and gloom, there are alternatives. (Spouse of a male driver living with dementia)

The visual presentation of the draft UK DDDA, including colour, layout, images and graphs, was highlighted as a favoured feature by stakeholders and ILWD. The clear simple layout, large font, basic language and limited text made the decision aid simple, easy to read and engaging. Participants commented:

The colourful easy to read layout. Simpleness. (Female, family member of individual living with dementia, 30–49 years)

It catches your eye right away—it's straight to the point. (Male, driver living with dementia, 72 years)

ILWD and their spouses revealed images that were not relevant, sad or negative should be replaced with more positive images related to driving. For example, one participant said:

[Image] looks like she has been told a relative has died or being told one of your dogs died. To me, it implies this lady has gone in and this lady has been told you can no longer drive. (Female, spouse of individual living with dementia)

##### Relevant and concise content

ILWD perceived that the content was balanced and useful and had the right amount of information. Valuable content to retain was information relating to warning signs and risks, alternatives to driving, and signposting to resources and contact details. For one individual living with dementia, the content on warning signs triggered them to start thinking about a time when they would stop driving:

And this [warning signs] made me really think seriously about stopping driving. I think this really covers the whole thing. (Female, retired driver with dementia, 82 years)

In contrast to the views of ILWD, some stakeholders felt the draft UK DDDA was too long, with too much information. One participant said:

I think it is too lengthy for someone with dementia to follow beginning to end. (Female, nurse, 30–49 years)

Conversely, despite the draft UK decision aid being regarded as too long, stakeholders also expressed a desire to include further information. Specific UK support details, driving regulations, obligations of drivers living with dementia and personal stories were suggested additions made by the participants. An occupational therapist suggested:

…full explanation of the DVLA [Driver and Vehicle Licensing Agency], notification process needs to be made clear first and foremost. The details of the local driving assessment centre would be good to include, those who have been allowed to continue could then get an independent report on their ability to drive safely. (Female, occupational therapist, 65–74 years)

##### Choice centred

Both ILWD and stakeholders felt that the UK DDDA would provide specific clinical practice benefits. These included assisting in raising the sensitive topic of driving with ILWD, having complex conversations about driving in a non‐threatening manner, assisting with the decision‐making process by encouraging drivers with dementia to consider their options and, most importantly, enabling the individual living with dementia to have agency with decisions about driving. Many of the stakeholders strongly felt that empowering the individual living with dementia to be central to decisions was a positive and crucial element:

…also enable them to make this decision themselves which evidence shows promotes the best outcome post‐cessation. (Female, dementia adviser, 30–49 years)

### 3.4 Stage 3: Amendments to create a final UK DDDA

Amending the draft UK DDDA from the suggested changes was grounded in four criteria: (1) facilitates a person‐centred approach; (2) conforms with the International Patient Decision Aids Standards^19^ and theoretical underpinnings of the Ottawa Decisional Support Framework and Ottawa Personal Decision Guide^44^; (3) follows guidelines for providing information to older people or people living with dementia^45^, ^46^ and (4) enhances applicability to a UK context.

The suggested changes from the online survey and interviews were exported into separate Microsoft Word documents, reviewed, discussed and consensus about their inclusion was reached by the research team. Data from interview transcripts were reviewed first so that the research team could understand the views and opinions of ILWD to ensure these views were central to decisions about amendments to the draft UK DDDA. When there were conflicting suggestions or no clear consensus between different participant groups in Part 1 and Part 2 or within the research team, priority was given to the views of ILWD. Once consensus was reached on the final amendments, the changes were made by a desktop publisher and the research team endorsed the final UK DDDA.

Most of the presentation features of the draft UK DDDA were considered relevant and therefore were retained in the final version of the UK DDDA. These features included the stepped approach, colours, layout, checklists and most images. The layout of the miscellaneous section was amended and combined with acknowledgements, references, author details and a smaller font size to reduce the overall length of the document. One image of a person crying was removed as it was not considered to encourage a supportive approach to conversations or decision‐making about driving. Some amendments were made to the content. Three personal perspectives, further information about fitness to drive guidelines, the labels of headings, additional contact details for the ‘Support’ and ‘Knowledge’ sections and a small planning activity as part of ‘The Next Step’ post‐decision were included. The order of driving options presented to the reader was rearranged to improve clarity. Of the four driving options, one was renamed from ‘drive less’ to ‘continue driving with changes’ to reflect the range of self‐regulation strategies that could be incorporated into changes to driving behaviour.

## 4 DISCUSSION

Developing decision aids from the outset can be prohibitive because of cost and time constraints.^26^ Culturally adapting existing decision aids can therefore be a cost‐effective and efficient alternative as identified in prior studies when adapting health interventions.^30^ This study highlights that, by including the views of end users in the adaptation process, the decision aid is acceptable to and purposefully designed for ILWD. The views of ILWD and their support networks informed the final version of the UK DDDA. We encourage researchers who develop decision aids for ILWD to consider adopting recruitment methods that are flexible, multiple and congruous to these individuals' needs to ensure the views of this group are obtained. The use of interviews provided a way to gain valuable insight into the views of ILWD about the draft UK DDDA which were otherwise limited by the use of an online survey.

**Figure 3:**
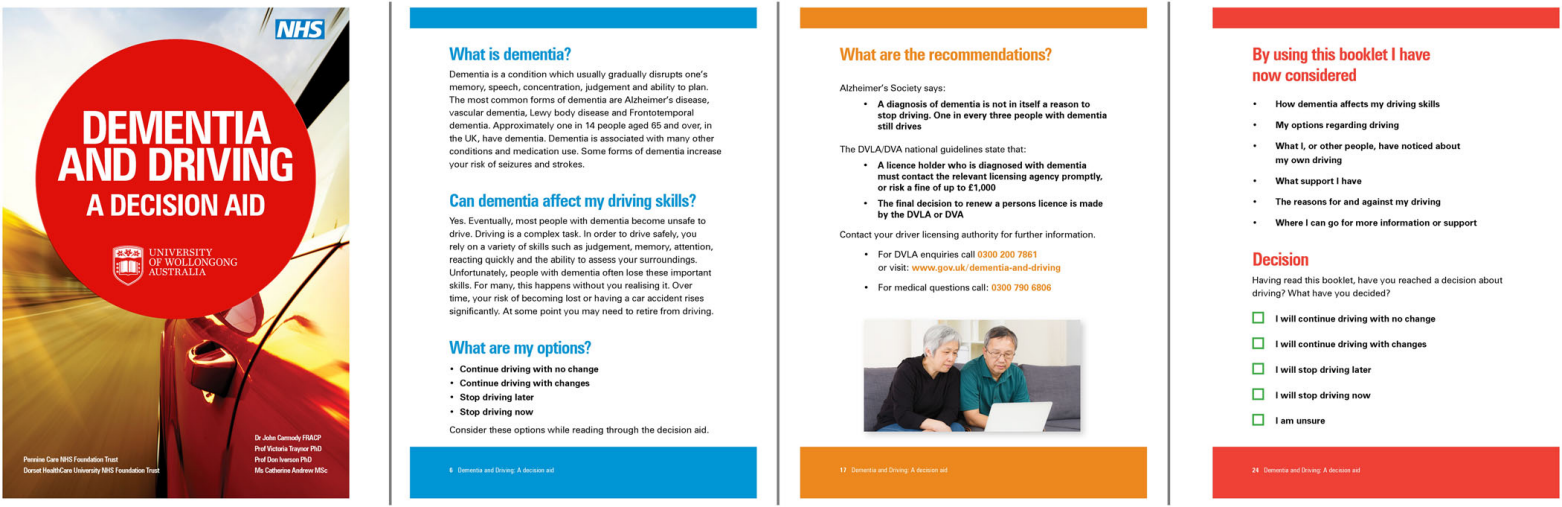
Preview of UK dementia and driving decision aid. (Images used under licence from Shutterstock.com).

Guidance for culturally adapting decision aids is a developing research area. Consistent with guidelines developed for culturally adapting health interventions, this study had a planned approach and included various stakeholder views in the adaptation process.^30^ Distinguishing the views of ILWD from those of other stakeholders, such as healthcare professionals, ensured a person‐centred approach was central to any changes made to the UK DDDA. The draft UK DDDA was overwhelmingly positively received by ILWD, family members and healthcare professionals (see Figure 3 for a preview of pages from the decision aid).

Stakeholder responses indicated the UK DDDA would be useful to family members and a diverse range of healthcare professionals, including nurses, general practitioners, dementia support workers, dementia advisers and psychologists, to assist them in supporting drivers living with dementia. There were various perceived benefits identified in this study that support previous findings reporting positive health outcomes for culturally enhanced health behavioural interventions.^31^ Assisting with initiating the sensitive topic of driving with ILWD, enabling the individual to have agency in decisions about driving and assisting with collaborative decision making were highlighted. A sense of control, empowerment, enhanced engagement, collaboration and communication were promoted by the inclusion of specific features. These benefits can be mapped to the basic psychological needs of self‐determination theory; competence, autonomy, and relatedness which are considered important to achieving a sense of well‐being.^47^ However, ‘relatedness’, identified as enhancing communication and collaboration in this study, may be attributed to the benefits of using decision aids rather than cultural adaptation.^25^

These benefits also align with a person‐centred process to care and decision making; open dialogue to enhance collaboration and fostering autonomy and self‐determination,^15^ which are valued by older people in decisions about driving.^16^ Both the format (which was structured and balanced) and the interactive nature of the decision aid through the provision of choice‐centred options were highly valued and perceived as key to empowering ILWD to engage with the decision process.

The findings highlight other important features of a decision aid for ILWD, including supportive messaging, appealing design and concise and plain English content. These findings are theoretically and empirically supported. For example, older people more broadly, and those in this study, are likely to be more receptive to positive over negatively framed information.^48^ Positive framing and dialogue are supported approaches for family members when initiating early conversations about driving with older people more generally.^49^ However, given international decision aid standards require decision aids to provide balanced content, presenting the decision aid in an appealing supportive format such as through colour, images and simple layout, could enhance engagement by ILWD.

Consequently, so that the UK DDDA was relevant and appropriate to the drivers living with dementia in the UK, some amendments to the draft were made from the findings. Additional localised contact details, content on legislative requirements, a planning activity, personal stories and amendments to options, grammar and images were made. The support of end‐users for the inclusion of personal stories in the decision aid builds upon prior studies reporting stories can be beneficial by way of increased knowledge and changes in intentions and attitudes.^50^ Many features were retained including the colours, layout, font size, signposting, steps, space for personalised responses and checklists which created an appealing interactive and individualised decision aid.

### 4.1 Practice implications

This study contributes to the limited decision aids for ILWD that include their views in the cultural adaptation process. We provide a case study as an example for future research involving the cultural adaption of decision aids for ILWD. Engaging the views of ILWD ensured the acceptability and authenticity of the final UK DDDA to meet the needs of drivers living with dementia who reside in the UK. The crucial features that were considered important inclusions in the decision aid identified by stakeholders in this study are generic, such as a structured, interactive format, supportive messaging and appealing presentation. For this reason, these features should be considered when developing decision aids for ILWD on other social and health topics. Further studies are required to test if the inclusion of the key features identified in this study impact engagement with the decision making process or shared decision making.

### 4.2 Limitations

Limitations of this study include the provision of a gift card as reimbursement for time to. This may have led to ILWD responding to the interviews according to what they felt the researcher wanted to hear. Additionally, individuals with early‐onset dementia were not represented in this study. These individuals might have different views, needs and challenges with the transition to not driving and therefore it is unclear whether the UK DDDA is useful or acceptable to this group. Furthermore, we did not explicitly seek participants' views on the potential barriers to engaging with the decision aid which might have provided further insights into how the decision aid could be enhanced. Most of the participants were from England and therefore the findings cannot be generalised to ILWD or healthcare professionals living in other countries within the UK. Future studies will require testing of the UK DDDA with a larger sample to understand the effects on knowledge and decisional conflict and to explore the usefulness of the decision aid to UK healthcare professionals in the clinical practice setting.

## 5 CONCLUSION

The UK DDDA provides a practical resource to guide decisions and conversations about driving for the increasing number of drivers living with dementia and their support networks. Interactive features and personalised design elements were perceived to facilitate a person‐centred approach and ensure ILWD have agency with driving decisions. Supportive messaging and appealing presentation features were valued elements of the decision aid. The findings of this study suggest that the UK DDDA will be useful and engaging to ILWD and the diverse range of healthcare professionals who provide support to ILWD when discussing and making decisions about driving.

## AUTHOR CONTRIBUTIONS

**Nadine Veerhuis**: Study design; analysis and interpretation; draft manuscript preparation; writing—reviewing and editing. **Alessandra Merizzi**: Conceptualisation; study design; data acquisition; analysis and interpretation; draft manuscript preparation; writing—reviewing and editing. **Stephanie Papoulias**: Data acquisition; analysis and interpretation; writing—reviewing and editing. **Claire Bradbury**: Data acquisition and interpretation; writing—reviewing. **Kathy Sheret**: Data acquisition and interpretation; writing—reviewing. **Victoria Traynor**: Conceptualisation; study design; resources; interpretation; writing—reviewing and editing; funding acquisition. All authors approved the final manuscript.

## CONFLICT OF INTEREST STATEMENT

The authors declare no conflict of interest.

## ETHICS STATEMENT

Ethical approval to undertake the study was provided by the UK National Research Authority (255012) and the University of Wollongong Human Research Ethics Committee (2019/03735) before data collection commenced.

## Supporting information

Supporting information.

## Data Availability

The data that supported the findings of this study are available from the corresponding author upon reasonable request.
