# Supplementary material for: ‘It is empowering and gives people dignity in a very difficult process’: A multistage, multimethod qualitative study to understand the views of end users in the cultural adaptation of a dementia and driving decision aid
Source: Health Expect. 2024 Mar 18;27(2):e14006. doi: 10.1111/hex.14006 (PMC10945392; doi:10.1111/hex.14006)
Supplement: Supplementary file 1 — Supporting information. [file HEX-27-e14006-s001.pdf]

Supporting Information Materials

Supporting Information Materials: Appendix A

COREQ (COnsolidated criteria for REporting Qualitative research) Checklist

A checklist of items that should be included in reports of qualitative research. You must report the page number in your manuscript where you consider each of the items listed in this checklist. If you have not included this information, either revise your manuscript accordingly before submitting or note N/A. (Page numbers from original manuscript submission).

Topic	Item No.	Guide Questions/Description (For interviews)	Reported on Page No.
**Domain 1: Research team and reflexivity**			
Personal characteristics			
Interviewer/facilitator	1	Which author/s conducted the interview or focus group?	9
Credentials	2	What were the researcher's credentials? E.g. PhD, MD	9
Occupation	3	What was their occupation at the time of the study?	9
Gender	4	Was the researcher male or female?	N/A
Experience and training	5	What experience or training did the researcher have?	9
Relationship with participants			
Relationship established	6	Was a relationship established prior to study commencement?	6
Participant knowledge of the interviewer	7	What did the participants know about the researcher? e.g. personal goals, reasons for doing the research	9
Interviewer characteristics	8	What characteristics were reported about the inter viewer/facilitator? e.g. Bias, assumptions, reasons and interests in the research topic	6, 9
**Domain 2: Study design**			
Theoretical framework			
Methodological orientation and Theory	9	What methodological orientation was stated to underpin the study? e.g. grounded theory, discourse analysis, ethnography, phenomenology, content analysis	6
Participant selection			
Sampling	10	How were participants selected? e.g. purposive, convenience, consecutive, snowball	8
Method of approach	11	How were participants approached? e.g. face-to-face, telephone, mail, email	8-9
Sample size	12	How many participants were in the study?	13
Non-participation	13	How many people refused to participate or dropped out? Reasons?	11
Setting			
Setting of data collection	14	Where was the data collected? e.g. home, clinic, workplace	9
Presence of non-participants	15	Was anyone else present besides the participants and researchers?	14
Description of sample	16	What are the important characteristics of the sample? e.g. demographic data, date	13
Data collection			
Interview guide	17	Were questions, prompts, guides provided by the authors? Was it pilot tested?	11
Repeat interviews	18	Were repeat interviews carried out? If yes, how many?	11
Audio/visual recording	19	Did the research use audio or visual recording to collect the data?	11
Field notes	20	Were field notes made during and/or after the interview or focus group?	11
Duration	21	What was the duration of the interviews or focus group?	11
Data saturation	22	Was data saturation discussed?	12
Transcripts returned	23	Were transcripts returned to participants for comment and/or	11

Topic	Item No.	Guide Questions/Description	Reported on Page No.
		correction?	
**Domain 3: analysis and findings**			
Data analysis			
Number of data coders	24	How many data coders coded the data?	11
Description of the coding tree	25	Did authors provide a description of the coding tree?	N/A
Derivation of themes	26	Were themes identified in advance or derived from the data?	12-13
Software	27	What software, if applicable, was used to manage the data?	11
Participant checking	28	Did participants provide feedback on the findings?	11
Reporting			
Quotations presented	29	Were participant quotations presented to illustrate the themes/findings? Was each quotation identified? e.g. participant number	13-18
Data and findings consistent	30	Was there consistency between the data presented and the findings?	13-18
Clarity of major themes	31	Were major themes clearly presented in the findings?	15
Clarity of minor themes	32	Is there a description of diverse cases or discussion of minor themes?	15

Developed from: Tong A, Sainsbury P, Craig J. Consolidated criteria for reporting qualitative research (COREQ): a 32-item checklist for interviews and focus groups. *International Journal for Quality in Health Care*. 2007. Volume 19, Number 6: pp. 349 – 357

Once you have completed this checklist, please save a copy and upload it as part of your submission. DO NOT include this checklist as part of the main manuscript document. It must be uploaded as a separate file.

Supporting Information Materials: Appendix B

DEVELOPTOOLS Reporting Checklist.

**Item**	**Explanation**	**UCD-11 Measure Scoring Yes=1/No=0**	**Reporting Checklist Additional Information**
**Preprototype involvement**			
1.Were potential users (patients, caregivers, family and friends, surrogates) involved in any steps to help understand users	Such steps could include various forms of user research, including formal or informal needs assessment, focus groups, surveys, contextual inquiry, ethnographic observation of existing practices, literature review in which users were involved in appraising and interpreting existing literature, development of user groups, personas, user profiles, tasks, or scenarios, or other activities.	1	Individuals living with dementia who were members of a support group voiced a desire to develop a UK dementia and driving decision aid (DDDA) after viewing an Australian dementia and driving decision aid (DDDA). Members of the support group highlighted the lack of clarity about driving licence responsibilities, the licensing renewal process, and the lack of information and support when driving retirement was imminent.
2. Were potential users (patients, caregivers, family and friends, surrogates) involved in any steps of designing, developing, and/or refining a prototype?	Such steps could include storyboarding, reviewing the draft design or content prior to starting to develop the tool, and designing, developing, or refining a prototype.a	0	Healthcare professionals only were involved in the development of the draft UK DDDA
**Iterative responsiveness**			
3.Were potential users (patients, caregivers, family and friends, surrogates) involved in any steps intended to evaluate prototypes of the tool or final version of the tool?	Such steps could include feasibility testing, usability testing with iterative prototypes, pilot testing, a randomized controlled trial of a final version of the tool, or other activities.	0	Evaluation of the decision aid has not yet occurred and was not part of this study
4.Were potential users (patients, caregivers, family and friends, surrogates) asked their opinions of prototypes of the tool or a final version of the tool in any way?	For example they might be asked to voice their opinions in a focus group, interview, survey, or through other methods.	1	Using interviews, individuals living with dementia and some of their spouses reviewed and provided feedback on a draft UK DDDA.<br>Using an online survey, family members, individuals living with dementia and other professionals reviewed and provided feedback on the draft UK DDDA. Eleven individuals and six of their spouses participated in interviews<br>24 family members, individuals living with dementia and other professionals completed an online survey.
5.Were potential users (patients, caregivers, family and friends, surrogates) observed using the tool in any way?	For example they might be observed in a think-aloud study, cognitive interviews, through passive observation, logfiles, or other methods.	0	The decision aid was provided to end users prior to the interviews and no observations in a clinical setting occurred.
6.Did the development process have 3 or more iterative cycles?	The definition of a cycle is that your team developed something and showed it to at least 1 person outside the team before making changes in response to their reactions or feedback. Each new cycle leads to a version of the tool that has been revised in some small or large way.	1	This study was a cultural adaptation of the decision aid which had already been iteratively developed. The cultural adaptation cycle for this study was 2: Development of a draft with health professionals, then end-user review of the draft, and then a final UKDDDA developed.

7. Were changes between iterative cycles explicitly reported in any way	For example, the team might have explicitly reported them in a peer-reviewed paper or in a technical report. In the case of rapid prototyping, such reporting could be, for example, a list of design decisions made and the rationale for the decisions.	1	The main amendments made to create a draft and the final UK DDDA are reported in the manuscript under Results, Stage 1 and Stage 3
**Other expert involvement**			
8. Were health professionals asked their opinion of the tool at any point?	Health professionals could be any relevant professionals, including physicians, nurses, allied health professionals, and so on. These professionals are not members of the research team. They provide care to people who are likely users of the tool. Asking their opinion means simply asking for feedback, in contrast to, for example, observing their interaction with the tool or assessing the impact of the tool on health professionals' behavior.	1	Health professional opinions were sought using e-mail for pre-prototype and an online survey to review the draft.
9. Were health professionals consulted before a first prototype was developed?	Consulting before a first prototype means consulting prior to developing anything. This may include a variety of consultation methods.	1	Healthcare professionals contributed to developing a draft UK DDDA by reviewing and amending the Australian DDDA. Individual feedback and amendments were provided on a PDF.
10. Were health professionals consulted between initial and final prototypes?	Consulting between initial and final prototypes means some initial design of the tool was already created when consulting with health professionals	1	Healthcare professionals reviewed and provided feedback on the draft UK DDDA through an online survey
11. Was an expert panel involved?	An expert panel is typically an advisory panel composed of experts in areas relevant to the tool if such experts are not already present on the research team (e.g., plain language experts, accessibility experts, designers, engineers, industrial designers, digital security experts). These experts may be health professionals, but not health professionals who would provide direct care to end users.	0	
**Additional elements in DEVELOPTOOLS Reporting Checklist**			
12. Was a formal advisory panel of users involved?	Such formal panels could be existing panels or they could be assembled for the project	0	
13. Were users (patients, caregivers, family and friends, surrogates), health professionals, and other relevant stakeholders involved as members of the research team?	User involvement on the research team implies that users had some level of decisional authority in the research plan. Similarly, health professional involvement implies that health professionals had some level of decisional authority in the research plan.	1	The research team consisted of psychologists and nurses who worked in memory clinics and would use the decision aid in clinical practice.
14. Were members of populations marginalized by social norms and policies involved?	Populations who have been marginalized by social norms and policies are social groups with a higher risk of health problems.24 These groups include but are not limited to people who are poor, discriminated against, stigmatized, marginalized, or disenfranchised due to psychological, physical, sensory or cognitive characteristics (e.g., mental illness, low literacy, disability), socioeconomic or sociocultural characteristics (e.g., education, income,	1	Individuals living with dementia participated in interviews and an online survey

	race/ethnicity, language, gender identity, sexual orientation, immigration status), or for other reasons (e.g., alcohol or drug dependencies).		
15. How many users (patients, caregivers, family and friends, surrogates) and health professionals were involved in total and of each type?	People should be reported according to group	1	Healthcare professionals or professional carers (external pre-prototype and online survey) N= 81<br>Family members or friend (interviews and online survey) N=16<br>Individuals living with dementia (interviews and online survey) N=14<br>Other support networks (online survey) = 11
16. Does the tool have a defined purpose?	The tools purpose may be to support shared decision making, to enable a person to accomplish a physical or cognitive task, to support self-management, or other purposes.	1	The decision aid has various purposes which are conveyed explicitly in the aid but also implicitly based on empirical and theoretical evidence underpinning the aid.<br>To enhanced knowledge and decisions about driving and alternative mobility options.<br>Facilitate early planning about changes to driving behaviour and driving retirement<br>Facilitate conversations and shared decision making about driving
17. Is the tool intended to be used in a particular context?	Tools may be intended to be used at home at any time, in a clinic during a consultation, or in other place/time contexts.	1	The decision aid is intended to be used at home, in the clinical practice setting, in community care situations, individually and as part of shared decision making.<br>Situations may include after diagnosis of dementia or during fitness to drive assessments.
18. Were any methods used to facilitate sharing of perspectives between groups	For example, workshops involving users (patients, families, caregivers, surrogates), health professionals, researchers, and other stakeholders may be used for this purpose.	NA	
19. Were users (patients, caregivers, family and friends, surrogates) involved from the outset of the project?	Users may be involved from the very beginning of a project to, among other things, help establish the purpose of the patient decision aid, its audience, and the scope of its content.	1	Concerns of individuals living with dementia as part of a support group instigated the international collaboration.
20. Were translation and cultural adaptation used to render the patient decision aid available to users across languages and cultures?	For example a patient decision aid might be developed in 1 language, then translated into 1 or more other languages and culturally adapted and validated to ensure it is acceptable to members of other cultures.23,b	1	No language translation was required. Cultural adaptation from Australian to United Kingdom.

NA, Not Applicable.

aActivities like think aloud and other evaluative exercises are considered prototype development activities (item 2) if they occur during rapid cycles of development (e.g., a codesign workshop) and if the users are involved in interpreting the data. If, on the other hand, users are simply shown the prototype and asked to think aloud or asked their opinions, this is considered a step intended to evaluate the tool (item 3).
bTranslation of a decision aid from 1 language (e.g., English) to another without careful consideration of cultural factors and needed adaptations is strongly discouraged
Adapted from: (Witteman et al., 2021, p746-48)

Supporting Information Materials: Appendix C

Online Survey Questions

GENERAL INFORMATION

What is your country of residence?

- England
- Northern Ireland
- Scotland
- Wales
- Other

Please indicate your gender

- Male
- Female
- Other (please specify)

Please select your age

I am …?

- An individual living with memory loss or a dementia
- A family member or friend of an individual living with memory loss or a dementia
- Professional carer/ practitioner
- Other (please specify)

PROFESSION

Which of the following best describes your role?

- Dementia Advisor Dementia specific worker Enrolled Nurse
- Family Physician Geriatrician Gerontological Nurse Neurologist
- Nurse Practitioner
- Occupational Therapist
- Personal Care Worker
- Physiotherapist
- Psychologist
- Registered Nurse
- Social Worker
- Other (please specify)

DEMOGRAPHIC INFORMATION (ILWD)

- What are your living arrangements?
- With spouse/ partner in family home
- With another family member/ friend in family home
- Alone in family home
- Retirement village/ Independent living centre
- Hostel/ supported accommodation
- Nursing home
- Other (please specify)

What is my highest level of schooling?

- Primary school
- Lower secondary school
- Upper secondary school
- Associate degree
- Bachelor degree
- Masters university degree
- Doctoral (PhD) degree
- Other (please specify)

I am a current driver?

- Yes
- No

If yes, please indicate how many years you have been driving

This is how often I drive…

- Less than once a week
- Once a week
- 2-6 times per week
- Once a day
- More than once a day
- Other (please specify)

My access to public transport is …

- Very good
- Good
- Reasonable
- Poor
- Very poor
- Not sure (please specify)

The following survey questions relate directly to a UK version of the Dementia and Driving Decision Aid booklet originally developed for consumers and carers living in Australia. Please read the booklet before answering the questions. You might find it useful to print out the booklet before answering these questions. If you have not already downloaded a copy, the booklet can be found on a web page: CLICK HERE TO ACCESS THE UK DEMENTIA AND DRIVING DECISION AID BOOKLET. (Please note: depending on your internet speed downloading the booklet may take a few minutes. The booklet will open in a new window).

TELL US YOUR VIEWS ABOUT THE BOOKLET

What did you like about the booklet?

- No comment
- I liked…. Please add comments below

What would you change about the booklet?

- No comment
- Yes, I would make changes. Please suggest changes in the space below

We would value your suggestions and comments to specific sections of the booklet.

- Yes, I would like to continue

- No, I would like to go to the end of the survey

The following questions were presented to participants who were willing to review each page of the decision aid. The following response options were presented:

- Yes
- No
- Not sure

If yes, what alternate wording would you suggest?

STEP 1: TO HELP CLARIFY YOUR DECISION

The following four questions relate to pages 6-9 of the booklet?

Would you change the wording or information to the question: " What is dementia"? (page 6)

- Would you change the wording or information to the question: "Can dementia affect your driving skills"? (page 6)
- Would you change the wording or information to the question: "What are your options"? (page 6)
- Would you change the wording or information to the question: "How far along are you with making a decision about driving?" (page 7)
- Would you change the wording or information provided to the question: "How will you cope without your car?" (page 7)
- Would you change the wording or information to the question: "Have you noticed any warning signs of unsafe driving?" (page 8)
- Would you change the wording or options to the question: "How often do you experience any of these warning signs?" (page 9)

STEP 2: WHAT DO YOU NEED TO MAKE YOUR DECISION?

The following four questions are related to pages 11-12 in the booklet.

- Would you change the wording to the questions relating to: "Support"? (page 11)
- Would you change the wording to the questions relating to: "Knowledge"? (page 11)
- Would you change the wording to the question on: "Values" (page 12)
- Would you change the wording to the question on: "Certainty"? (page 12)

STEP 3: WEIGHING YOUR OPTIONS

The following questions relate to pages 14-19 of the booklet.

- Would you change any of the wording or information for the questions relating to: "Support" (page 14)
- Would you change any of the wording for the information provided relating to: "Knowledge" (page 15)
- Would you change any of the wording or options provided in: "What is the most important reason for you to continue driving" (page 18)
- Would you change any of the wording or options provided in: "What is the most important reason for you to stop driving?" (page 19)

STEP 4: WHAT NEXT?

The following survey questions relate to pages 21-25 of the booklet

- Would you change any of the wording or information provided in the section on: “Support" (page 21)
- Would you change any of the wording or information provided in the section on: "Knowledge" (page 22)

- Would you change any of the wording or information provided relating to: "Values" (page 23)

FINAL COMMENTS AND SUGGESTIONS

Do you have any final suggestions or comments to make in general about the booklet?

- Yes
- No
- Not sure

If yes, please provide details.

Would you like to receive notice when the U.K booklet is available?

- Yes
- No
